# Systematic review—Time to malignant transformation in low-grade gliomas: Predicting a catastrophic event with clinical, neuroimaging, and molecular markers

**DOI:** 10.1093/noajnl/vdab101

**Published:** 2021-07-27

**Authors:** Zabina Satar, Gary Hotton, George Samandouras

**Affiliations:** 1 University College London, Queen Square Institute of Neurology, London, UK; 2 University College London Hospitals NHS Trust, Victor Horsley Department of Neurosurgery, The National Hospital for Neurology and Neurosurgery, London, UK; 3 The National Hospital for Neurology and Neurosurgery, Queen Square, UK; 4 North Middlesex University Hospital, London, UK

**Keywords:** clinical marker, low-grade glioma, malignant transformation, molecular marker, radiological marker

## Abstract

**Background:**

Despite an initially indolent course, all WHO grade II, LGGs inevitably transform to malignant, WHO grades III and IV, without current curative options. Malignant transformation (MT) remains unpredictable with limited prognostic markers to steer timing of interventions. The aim of this study was to review and assign predictive value to specific clinical, molecular, and radiological markers impacting MT, thereby justifying timely therapeutic interventions.

**Methods:**

Searches of MEDLINE, Embase, and Cochrane databases were conducted from inception to April 28, 2021 and outputs were analysed in accordance with PRISMA protocol.

**Results:**

From an initial 5,032 articles, 33 articles were included, totalling 5672 patients. Forty-three prognostic factors were registered to significantly impact MT. These were categorised as 7 clinical; 14 neuroimaging; 8 biological/molecular; 3 volumetric; 5 topological; 3 histological; and 3 treatment-related. Following analysis, 10 factors were highlighted: the pre-operative prognosticators were 1. presentation with epileptic seizures; 2. VDE > 8 mm/y; 3. VDE > 4 mm/y; 4. rCBV > 1.75; 5. PTV ≥ 5 cm (65 ml); 6. PTV ≥ 100 ml; and 7. cortical involvement. The post-operative prognosticators were: (1) IDH-wt, (2) TP53 mutation, and (3) temozolomide monotherapy.

**Conclusions:**

The management of LGGs remains controversial, as conservative and invasive treatment may be associated with MT and impaired quality of life, respectively. Our review indicates that MT can be predicted by specific metrics in VDE, PTV, and rCBV, alongside cortical involvement. Additionally, patients with IDH-wt tumours TP53 mutations, or receiving TMZ monotherapy are more likely to undergo MT. Our data may form the basis of a predictive scoring system.

**Key Points**

- MT of LGGs remains unpredictable with limited prognostic markers.
- We identified 43 distinct markers significantly impacting MT and ten were highlighted.
- Routine neuroimaging and clinical data, may identify higher risk cohorts justifying early intervention.

**Importance of the Study**

Malignant transformation of LGGs remains largely unpredictable with prognostic markers limited. No practice guidelines or direction for timing of interventions presently exist. Our aim was to systematically review, identify and assign prognostic weight to specific clinical, molecular, and radiological markers impacting MT in LGGs.

 Analysis of the 33 qualifying articles, totalling 5672 patients, identified 43 prognosticators significantly impacting MT and 10 prognosticators were highlighted: (1) presentation with epileptic seizures, (2) VDE > 8 mm/y, (3) VDE > 4 mm/y, (4) rCBV > 1.75, (5) PTV ≥ 5 cm (65 ml), (6) PTV ≥ 100 ml, (7) cortical involvement, (8) IDH-wt, (9) TP53 mutation, and (10) TMZ monotherapy.

 To our knowledge, this is the first systematic review aiming to prognosticate categorical factors, based on both post-, and more importantly, pre-operative factors. Our results indicate that neuroimaging and clinical data, may identify higher risk LGG patients, justifying early invasive interventions. Although, not a primary aim, a scoring system may be introduced.

World Health Organisation (WHO) grade II supratentorial low-grade gliomas (LGGs), are generally slow-growing, infiltrating primary brain tumours, however, they can also constitute an ultimately and uniformly fatal disease of, usually, fully functioning young adults presenting with a degree of neuro-cognitive decline (mean age 41 years) with overall survival (OS) averaging between 5 and 15 years.^1–6^ Patients harbouring LGGs can continue to work and perform well for considerable periods of time, often years, without treatment and minimal radiological progression, providing a false sense of security, as the tumours progress unpredictably but, invariably, to malignant variants.^7^

LGGs compose a heterogenous group of tumours with distinct molecular, clinical, and histological features^8^ with an incompletely understood natural history ranging from indolent to biologically aggressive behaviour, resulting ultimately in neurological decline and death secondary to malignant transformation (MT).^3,4,9–13^ In addition, compared to its 2007 predecessor, the 2016 update of the WHO classification of CNS tumours has included molecular features with histology to create an integrated diagnosis.^14^ Despite the discovery of several biomarkers such as mutations in the isocitrate dehydrogenase (IDH) genes 1 and 2, in combination with ATRX loss or 1p/19q co-deletion, and very recently, the additional validation of the relevance of CDKN2A/B homozygous deletions, representing remarkable milestones in stratifying LGGs, an objective, reproducible and prognostically relevant classification remains elusive.^8,15^

The reported frequency of LGG MT ranges from 25% to 72% in the published literature^9,16^ highlighting the variability of MT and potential multifactorial contributions to a catastrophic event. The vast majority of LGG studies focus on general prognostic factors of survival, mainly devised before the 2016 update of the WHO classification on CNS tumours, including eloquent location, Karnofsky performance score (KPS) > 80, age < 50 years, or tumour diameter < 4 cm, confirmed with Cox proportional hazards modelling or, alternatively, loosely defined progression outcomes which do not necessarily equate to MT.^17^

Understanding the non-linear, unpredictable process of MT is critical to planning therapeutic interventions, as resective surgery or radiotherapy may be associated with neurological morbidity in a typically highly functioning cohort of patients.^18^ Although a number of factors have been reported to be associated with MT including preoperative tumour size,^9,19^ velocity of diametric expansion (VDE),^20,21^ astrocytoma histology,^9^ and extent of resection^14,22,23^ they have not yet been studied in a systematic way.

In the current systematic review, employing a strict definition of MT and minimising the biases of false positive results, we aim to comprehensively and critically appraise the modern literature extracting all relevant metrics of MT, thereby presenting the first systematic review of the literature, producing clinical, molecular and radiological factors predictive of MT in LGGs.

## Methods

The systematic review was conducted in accordance with the PRISMA (Preferred Reporting Items for Systematic Reviews and Meta-Analyses) guidelines, Cochrane Methods Prognosis Group's review template, and Riley's guide to a systematic review and meta-analysis of prognostic factor studies.^24–26^

### Eligibility Criteria

#### Study type

Prospective and retrospective original studies investigating prognostic factors for MT in LGGs, written in English, were included. Studies investigating prognostic factors in cell lines/xenograft models were excluded. Case reports, reviews, conference abstracts, and editorials were also excluded.

#### Participants

Age threshold was set to adult and adolescent patients (≥15 years of age) reflecting a dichotomy in paediatric and adult services. Mixed adult and child (<15 years) populations were included if adult cohorts could be analysed separately. Diagnoses included were WHO grade II gliomas (oligodendroglioma, astrocytoma, or oligoastrocytoma). Studies with pilocytic astrocytomas, gangliogliomas, and ependymomas were excluded as typically belong in WHO grade I tumours.^27–29^

#### Definitions and outcomes

The outcomes of MT and reported prognosticators were recorded. The aim was identification of high-impact MT markers with potential of validation and further assessment in future studies.

To account for disparities in MT definitions in the literature and ensure reproducible MT interpretations, a strict definition was applied, as the progression of a WHO grade II tumour (diffuse astrocytoma, oligodendroglioma) to a higher WHO grade III or IV tumour (anaplastic astrocytoma, anaplastic oligodendroglioma, or glioblastoma) confirmed histologically by surgical specimen or radiologically as new/increased contrast enhancement on T1 weighted MRI scan after the administration of gadolinium.

### Sources and Search Strategy

Electronic searches of Ovid MEDLINE, Embase, and Cochrane library databases were conducted from the inception of each database to April 28, 2021. Full details of the search protocols are provided in the Supplementary Methods.

### Selection Process, Study Records, and Data Management

All search results were assessed for eligibility. Initially, records were examined by title and abstract while in the second round, full text retrieval and review were employed. Qualifying studies were included in the systematic review. Results of the literature search were imported to EndNote X9 (Clarivate Analytics). Software was used to reduce data entry errors and to deduplicate search results. Any further duplicates found during screening were manually excluded. Data recorded in a Microsoft Excel spreadsheet included: author, publication year, and journal citation; patient inclusion and exclusion criteria; study design; study population; tumour details at diagnosis (tumour size, and histology); length of follow-up; prognostic factors investigated; rate of MT; significant association between factors of interest and MT; other key results; definition of MT adhered to by the study.

### Risk of Bias Assessment

The methodological quality of the studies was assessed using the Quality in Prognosis Studies (QUIPS) tool with a list of considerations.^30,31^ The criteria list was adjusted to establish criteria for follow-up and drop out percentage.^32^ Each criterion was scored with “yes,” “no,” or “don’t know,” which led to the overall scoring of low, moderate, or high risk of bias for the following 6 domains of potential biases: (1) study participation, (2) study attrition, (3) measurement of prognostic factors, (4) measurement and control for confounders, (5) measurement of outcomes, and (6) analysis (Supplementary Figure 1). All criteria were scored as follows: “yes” (Y) for adequate description of the criterion at issue and study meets the criterion; “no” (N) for lack of such; or “don’t know” (U) for insufficient information. The issues were taken together to form an overall judgement regarding each domain. A study was considered to be of high quality when the methodological risk of bias was rated as low or moderate across all of the 6 domains.

## Results

The electronic search yielded a total of 6009 abstracts. After deduplication, 4314 abstracts were screened by title and abstract for eligibility. In accordance with the inclusion criteria, 4637 records were excluded, and 323 publications were flagged for full-text review. Following a full-text assessment of each record, a total of 33 articles were identified for the final data extraction. Details outlining the reasons for exclusion and full flow of information, in accordance with the PRISMA format, are presented in Supplementary Figure 2.

In total, the 33 qualified articles reported data totalling 5672 patients. A total of 43 distinct, measurable prognostic factors were found to have a significant effect on MT and catalogued accordingly. To facilitate a systematic, quantitative analysis, prognosticators were grouped into 7 categorical domains as follows: *molecular/biological*, 8 prognosticators; *radiological/imaging*, 14 prognosticators; *clinical*, 7 prognosticators; *tumour volumetry*, 3 prognosticators; *topological/anatomical*, 5 prognosticators; *histological,* 3 prognosticators; *treatment-related,* 3 prognosticators (Supplementary Table 1). Prognosticators were also categorised as negative, or positive, when seen to promote, or impede, respectively, MT.

In addition, prognosticators were classified as pre-operative when no molecular, histopathological, or treatment-related data were available, and post-operative prognosticators when these data were available. Finally, prognosticators were classified as binary, when the presence or absence of a mutation or a clinical feature could characterize a specific factor, and categorical, when a prognosticator could be stratified within numerical thresholds of volume, size, or velocity.

### Clinical Domain

History of epileptic seizures^33,34^ in two studies and KPS ≥ 90^34^ were identified as positive prognosticators, while the 5 negative prognosticators were: (1) age > 45 years in two studies^33,35^, and >35 years in one study,^36^(2) male gender in two studies,^33,37^ (3) increased intracranial pressure (ICP) at diagnosis, in two studies,^33,38^ (4) neurological deficit at diagnosis in two studies,^33,34^ (5) duration of pre-operative symptoms >2 years in one study,^34^ and (6) KPS ≤ 70 in two studies.^33,35^

In a retrospective multi-institutional observational study involving 1509 patients, history of epileptic seizures at diagnosis was a statistically significant (*P* <.001), independent prognostic factor of delayed MT, with MT occurring from diagnosis at a mean time of 65.1 ± 54.8 and 39.5 ± 28.3 months, in subgroups with and without epileptic seizure at diagnosis, respectively. Interestingly, the same study reported that the probability of seizures was not affected by tumour volume or growth velocity, cortical location, and histological or molecular subtyping.^33^

### Radiological/Imaging Domain

Twelve negative prognosticators included (1) high regional cerebral blood flow (rCBV) in three studies,^39–41^ quantified as rCBV > 1.75 in one additional study,^42^ (2) velocity of diametric expansion (VDE) ≥ 8 mm/y^20,21,38^ with lower VDE of >3mm/y in one study,^41^ (3) volumetric difference between preoperative tumor volumes on T2- and T1-weighted MRI studies (∆VT2T1) > 30 cm,^3,43^ (4) presence of contrast enhancement on MRI/CT,^19,20,33,34,44^ (5) low apparent diffusion coefficient (ADC),^39^ (6) high normalised tCr (creatine/phosphocreatine) in magnetic resonance spectroscopy (MRS),^44^ (7) high mean and maximal choline/creatine ratio in MRS,^41^ (8) fluorescence with five-aminolevulinic acid (5-ALA),^45^ (9) tumour growth in 6 months interval scanning,^40^ (10) mean^18^F-FET (O-(2-^18^F-fluoroethyl)-L-tyrosine) uptake >1.1,^46^ (11) early tumour recurrence (within 2 years of primary surgery),^47^ and (12) high velocity radiological progression (time to definitive radiological change being ≤6 months).^47^

In a retrospective study of 380 patients, fast VDE (≥8 mm/y) was an independent prognostic factor, significantly associated with reduced malignant progression free survival (MFS), with the mean time to MFS being significantly longer in the slow subgroup (<8 mm/y) (mean, 119.2 months; range, 1–253 months) compared to the fast subgroup (mean, 41.4 months; range, 2–206 months; *P* < .001). Furthermore, MFS was significantly longer in patients with VDE < 4 mm/y than those with VDE ≥ 4 mm/y (*P* < .001). Likewise, patients with VDE ≥ 4 and <8 mm/y had significantly longer MFS than patients with VDE ≥ 8 mm/y (*P* < .001), and those with VDE ≥ 8 and < 12 mm/y had significantly longer MFS than with VDE ≥ 12 mm/y (*P* =.044). Of note, the VDE was significantly slower in tumors with 1p19q codeletion (*P* =.008) and with complete 1p deletion (*P* = .042) and was significantly faster in tumors with p53 overexpression (*P* = .003).^20^ Another study of 168 patients demonstrated that VDE ≥ 4 mm/y, VDE ≥ 8 mm/y, and VDE ≥ 12 mm/y all statistically conferred significantly shorter MFS compared to tumours with VDE < 4 mm/y (*P* < .001 for all 3 thresholds). Interestingly, subventricular zone (SVZ) involvement predicted high VDE in the same study: patients with SVZ involvement (7.16 ± 6.53 mm/y) had a significantly higher VDE than patients without (4.38 ± 5.35 mm/y) (*P* = .003).^21^

The effect of rCBV on MT was demonstrated in 4 studies. In a case-series prospective study of 63 patients, high rCBV (>1.75) was found to be independently associated with shorter MFS; *P* = .035). Additionally, the study showed that a threshold of rCBV = 1.742 was of optimal sensitivity (61.9%) and specificity (83.3%) in differentiating the group of patients with progression from the progression-free group.^42^ In another prospective study of 34 patients, the risk of MT was 1.75× higher per each additional standard deviation (SD) of rCBV at study entry (*P* = .01; SD = 0.6). Interestingly, in the subgroup of pure astrocytomas (*n* = 20) this effect was heightened as the risk of MT was 12.99× higher per SD of rCBV at study entry (*P* =.001; SD = 0.52).^40^ Finally, 2 further studies involving 78^39^ and 21 patients,^41^ reported high rCBV, defined as increased rCBV compared to the contralateral normal white matter, to have a negative prognostic effect on MT. A circumscribed tumour on MRI^46^ and local recurrence location^47^ had a positive prognostic effect.

### Molecular/Biological Domain

Seven factors were negative prognosticators and included: (1) IDH1 wild-type (wt),^37,38,45^ (2) p53 overexpression,^48,49^ (3) TP53 mutation,^50,51^(4) triple combination of IDH-mutated/*MGMT*-methylated/TP53 positive,^52^ (5) positive vascular endothelial growth factor (VEGF) staining,^35^ (6) microvessel density >7 microvessels,^35^ (7) Ki 67 labelling index over expression,^48^ and (8) intact 1p/19q.^18,37,53^ The positive and negative prognostic effects of 1p/19q codeletion were confirmed by different study groups and not by reverse assumptions.

In a retrospective cohort study of 486 patients, IDHwt tumours were significantly more likely to undergo MT than IDH mutated and 1p/19q co-deleted (IDHmut/codel tumours) (*P* < .001), with the 5-year estimates of MT delay of 82% for IDHwt and 92% for IDHmut/codel tumours. In the same study, patients with IDHwt tumours were at a 5.5-fold increased risk of MT.^37^ In a further study involving 74 patients, IDHwt was a significant poor prognostic factor for MT with MFS being significantly shorter in IDHwt tumors (39.0, 25.6–52.4 months) than in IDHmut tumours (64.6, 57.3–71.9 months) (*P* = .043) and was associated with around a 3-fold higher risk of MT.^45^

In a retrospective study involving 159 patients, positive TP53 mutation status (but not P53 overexpression) was the lone risk factor with respect to MT (*P* < .03).^50^ In a further study with 36 patients, TP53 was strongly and statistically associated with MT (*P* = .0344), specifically, 9 of 14 tumors (75%) harbouring TP53 mutations showed MT within 12 ± 75 months (median 37 months), compared to 9/22 tumors (41%) wild type TP53 tumours.^51^ Only 1p/19q co-deletion had a positive prognostic effect on MT.^18,20,54^

### Tumour Volumetry Domain

Larger pre-operative tumour volume (PTV) and size were a negative prognosticator in both pre-operative MRI scans, and post-operative residual volume, when surgery was performed, with a greater extent of resection^14^ having a positive prognostic effect. Initial tumour size of >3 cm^9,35^ and >5 cm^34,37^ were reported in two studies each, as negative MT prognosticators.

A retrospective study involving 191 patients, tumour size of the largest tumour dimension ≥3 cm was an independent factor associated with increased risk of MT and was found to have the greatest statistical significance compared to other parameters investigated in the study (*P* = .03). Specifically, tumours ≥ 3 cm had a 2.6-fold increase in undergoing MT.^9^ Another study with 74 patients, also found tumour size >3 cm to be statistically significant for freedom from MT (*P* < .05).^35^ In a retrospective study of 353 patients, tumour size ≥ 5 cm was a statistically significant, independent prognosticator for MT (*P* < .001), with 3.5-fold higher risk for tumours of this size.^37^ A further study of 148 patients revealed tumours > 5 cm (preoperative maximal diameter) to be a significantly associated with worsened MFS (*P* = .047).^34^

Tumour volume (TV) prognostication was reported in several studies but with three different thresholds of >20 ml^36^; >60 ml^53^; and ≥100 cm,^3,20,33^ all impacting on MT. In addition, association between larger TV (cm^3^) and MT was supported extensively in the literature but without setting specific thresholds.^22,42,43,55,56^

In a long-term retrospective study of 239 patients, pre-treatment tumour volume of >20 ml was significantly associated with increased risk of MT (*P* = .01).^36^ A study of 62 patients found tumour volume of >60 ml at diagnosis to be strongly predictive of increased MT risk (*P* < .001).^53^ Finally, a threshold of ≥100cm^3^ of tumour volume at diagnosis was demonstrated to significantly impact MT in 2 studies. The first study involving 1509 patients found this to be an independent prognostic factor for worsened MFS (*P* = .007),^33^ the second study, consisting of 380 patients also found this to be a significant factor (*P* = .008), associated with a 1.76-fold increase in MT risk.^20^ Five studies reported a statistically significant link between greater preoperative volume (cm^3^) and increased risk of MT, in a sum of 539 patients across all 5 studies.^22,42,43,55,56^

Studies reporting TV in post-operative settings either established a threshold of >30 ml,^53^ or did not quantify thresholds but still reinforced the association between larger post-operative TV and MT.^43,55^ Following surgical intervention, reports clearly supported the relationship between greater EOR (extent of resection), either in absolute post-operative TV volumes^22,43^ or in relation to original TV (EOR ≥ 90%).^47^ Similarly, gross total resection (GTR) conferred a positive prognostic effect.^9,33,34,38,54,57^ Furthermore, first line surgical resection compared to surveillance^20^ partial resection compared to biopsy^33,34^ and subtotal resection also compared to biopsy^33^ were all found to have a positive prognostic effect. Similarly, a postoperative residual tumour volume of ≤5 ml resulted in delayed MT compared to greater residual volumes.^58^ The converse was also confirmed in different studies, with watchful waiting compared to resection^59^ and smaller EOR^42^ having a negative prognostic effect on MT.

### Topological/Anatomical Domain

All the following five identified factors appeared to be associated with MT: (1) SVZ involvement,^21^ (2) parietal tumour location,^38^ (3) presence of cortical involvement,^33^ (4) eloquent tumour location,^22,34^ and (5) multilobar involvement.^53,56^

The presence of cortical involvement was shown to be an independent negative prognosticator for MT in a retrospective study of 1509 patients (*P* = .004).^33^ In a retrospective study involving 168 patients, SVZ involvement predicted a significantly shorter MFS (*P* = .033) and was independently associated with higher VDE (*P* = .003).^21^ The link between eloquent tumour location and MT was reported in 2 studies involving 148 patients and 216 patients, where it was shown to be significantly associated with worsened MFS (*P* < .001 and *P* = .006).^22,34^

### Histological Domain

Three histological subtypes were identified to be prone to MT: (1) fibrillary astrocytoma,^9,43^ (2) pure astrocytoma,^42,53^ and (3) gemistocytic astrocytoma.^39,53^ In a study of 190 patients fibrillary astrocytomas were significantly more likely to undergo MT than oligodendrogliomas or oligoastrocytomas, with the associated risk being 3.2-fold higher in these tumours (*P* = .003).^43^ In a further retrospective study involving 191 patients undergoing resection, fibrillary astrocytoma pathology was an independent negative prognostic factor for MT (*P* = .04).^9^

### Treatment-related Domain

Finally, treatment with chemotherapy (CT),^33^ radiotherapy (RT),^33,43^ and adjuvant chemo-radiotherapy^54^ all retarded MT. In accordance with these results, CT monotherapy (vs. RT followed by adjuvant CT)^37^ was found to have a negative prognostic effect on MT. However, in a retrospective study involving 472 patients, temozolomide (TMZ) monotherapy was a statistically significant and independent negative prognostic factor for MT (*P* = .08) when compared to patients who received both CT and RT. Interestingly, in the same study patients with IDHmut 1p/19qcodel tumours, which was the subgroup at lowest risk of MT, were more likely to be treated with adjuvant TMZ alone (*P* < .001).^37^

### Risk of Bias Assessment

According to QUIPS assessment tool, 26 of the 31 included studies were rated as high quality, meaning that the risk of bias was deemed as low or moderate across all 6 of the domains considered. Of the studies that were considered of low quality, 3 out of 4 of these were due to high risk of bias in the confounding factor domain. All studies had a low risk of bias in the study participation and outcome (i.e., definition and measurement of MT) domains. The results of the quality assessment are presented in Supplementary Figure 3.

## Discussion

Malignant transformation represents a critical and irreversible event in the course of LGGs, and the inability to predict its occurrence on a linear timeline renders timing of therapeutic interventions uncertain or, even, arbitrary. Studies balancing MT predictability, on preoperative data, are extremely sparse in the literature. To our knowledge, this is the first systematic review aiming to prognosticate categorical factors, based on both post-, and more importantly, pre-operative factors. In addition, recent molecular prognosticators render the group of LGGs challenging to precisely define. For example, IDH-wt mutations in previously defined WHO grade II gliomas tend to be treated as glioblastomas, although growing evidence suggests that this is an inhomogeneous group, that needs to be further stratified.^60,61^

**Figure 1.**
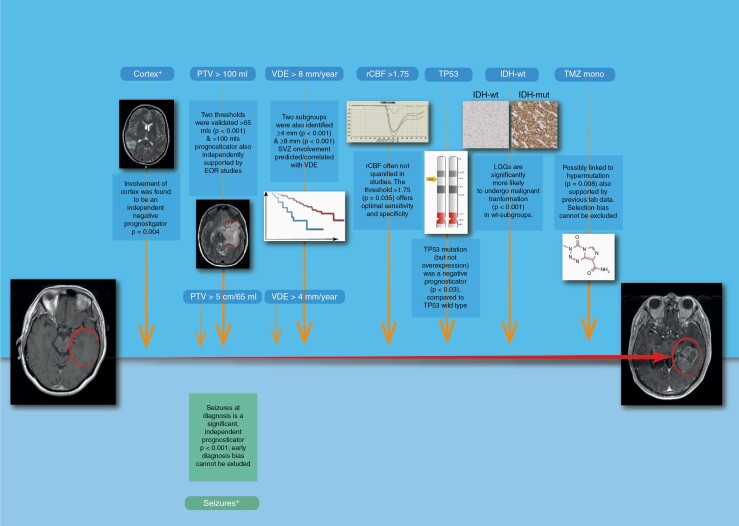
Summary of the 10 prognosticators of MT in LGGs. Demonstration of key nine negative (orange arrows) and one positive (green textbox) prognosticators, in low-grade glioma timeline, from initial presentation (far left) to malignant transformation in aggressive, gadolinium-enhancing forms (far right).

Based on review of 31 articles, out of 3808 reviewed abstracts, totalling 5193 patients, the current study has identified a total of 43 distinct, prognostic factors, spanning 7 categorical domains, with significant impact on MT in LGGs. The impact of the 43 factors was evaluated based on (1) statistical significance, (2) cohort size per study, (3) study quality, and (4) practical applicability, which led to the emergence of 10 prognostic factors, subdivided into 7 pre-operative, and 3 post-operative prognosticators, based on availability of histological and molecular analysis following surgical intervention. Five of the highlighted factors were described as binary (present or absent) and 5 were categorical, grouped by numerical thresholds. The pre-operative prognosticators were (1) presentation with epileptic seizures, (2) VDE > 8 mm/y, (3) VDE > 4 mm/y, (4) rCBV > 1.75, (5) PTV ≥ 5 cm (65 ml), (6) PTV ≥ 100 ml, and (7) cortical involvement. The post-operative prognosticators were (1) IDH-wt, (2) TP53 mutation, and (3) TMZ monotherapy (Table 1 and Figure 1). It is evident that two prognosticators, VDE and PTV, appear twice with different numerical thresholds, for reasons elucidated below.

**Table 1.** MT Prognosticator Table

Prognosticator	Number of Patients and *P*-values	References	Effect on MT	Comments
Pre-operative Prognosticators				
Epilepsy (B)	*N* = 1509; *P* < .001^33^ *N* = 148; *P* = .011^34^	Pallud et al.^33^ Gousias et al.^34^	D D	In a large retrospective multi-institutional observational study with 1509 patients, history of epileptic seizures at diagnosis was a statistically significant (*P* < .001), independent prognostic factor for delayed MT.^33^
VDE ≥ 8 mm/y (C)	*N* = 380; *P* < .001^20^ *N* = 168; P < .001^21^ *N* = 131; P < .001^38^	Pallud et al.^20^ Wen et al.^21^ Gozé et al.^38^	P P P	In 380 patients MPFS was significantly longer in the subgroup with VDE < 8 mm/y (median, 103 months; mean, 119.2 months; range, 1–253 months) than in the subgroup with VDE ≥ 8 mm/y (median, 35 months; mean, 41.4 months; range, 2–206 months; *P* < .001).^20^ In 168 patients, VDE ≥ 4 mm/y, VDE ≥ 8 mm/y and VDE ≥ 12 mm/y were all independently associated with shorter MFS (*P* < .001 for all).^21^ In 131 patients MFS was significantly longer in the VDE < 8 mm/y subgroup (median, 149 months; mean, 142) than in the VDE ≥ 8 mm/y subgroup (median, 46 months; mean, 56.2; *P* < .001).^38^ Within the VDE < 8 mm/y category, difference also appeared between VDE < 4 mm/y and VDE ≥ 4 mm/y (see below).
VDE ≥ 4 mm/y (C)	*N* = 168; *P* < .001^21^	Wen et al.^21^	P	VDE ≥ 4 mm/y was statistically significant for conferring shorter MFS in 168 patients with low-grade astrocytoma (*P* < .001).^21^
rCBV > 1.75 (C)	*N* = 63; *P* = .035^42^	Majchrzak et al. ^42^	P	In a case-series prospective study with 63 patients, high rCBV (>1.75) was independently associated with worse MFS; *P* = .035). A threshold of rCBV = 1.742 was of optimal sensitivity (61.9%) and specificity (83.3%) in differentiating the patients with progression and without.^42^
PTV ≥ 5 cm (65 ml) (C)	*N* = 353; *P* < .001^37^ *N* = 148; *P* = .047^34^	Tom et al.^37^ Gousias et al.^34^	P P	In a retrospective study of 353 patients, tumour size ≥ 5 cm was a statistically significant, independent prognosticator for MT (*P* < 0.001), with the risk being 3.5-fold higher for tumours of this size.^37^ In 148 patients, tumours >5 cm (preoperative maximal diameter) was significantly associated with worsened MFS (*P* = .047).^34^
PTV ≥ 100 ml (C)	*N* = 380; *P* = .008^20^ *N* = 1509; *P* = .007^33^	Pallud et al.^20^ Pallud et al.^33^	P P	Volume of ≥100 ml was independently and statistically associated with shortened MFS in 380 patients (*P* = .008).^20^ In 1509 patients, tumour volume of ≥100 ml was an independent factor for worsened MFS (*P* = .007).^33^
Cortical involvement (B)	*N* = 1509; *P* = .004^33^	Pallud et al.^33^	P	The presence of cortical involvement was an independent negative prognosticator for MT in a retrospective study of 1509 patients (*P* = .004).^33^
Post-operative Prognosticators				
IDH-wt (B)	*N* = 486; *P* < .001^37^ *N* = 131; *P* = .019^38^ *N* = 74; *P* = .043^45^	Tom et al.^37^ Gozé et al.^38^ Jaber et al.^45^	P P P	In a retrospective cohort study of 486 patients IDHwt tumours were significantly more likely to undergo MT than IDHmut/codel tumours (*P* < .001). The 5-year estimates of freedom from MT were 82% for IDHwt and 92% for IDHmut/codel tumours.^37^ In 131 patients, lack of IDH1 mutation was independently and significantly associated with shortened MFS (*P* = .019).^38^ In 74 patients, MFS was significantly shorter in IDHwt tumors (39.0, 25.6–52.4 months) than in IDHmut tumours (64.6, 57.3–71.9 months) (*P* = .003).^45^
TP53 mutation (B)	*N* = 159; *P* < .03^50^ *N* = 36; *P* = .0344^51^	Ständer et al.^50^ Ishii et al.^51^	P P	Positive TP53 mutation status (but not P53 overexpression) was the lone risk factor with respect to MT in a series with 159 patients.^50^ In 36 patients, TP53 was strongly associated with MT. Nine of 14 tumors (75%) harboring TP53 mutations showed MT within 12 ± 75 months (median 37 months), compared to 9/22 tumors (41%) wild type TP53 tumours.^51^
TMZ monotherapy (B)	*N* = 472; *P* = .008^37^	Tom et al.^37^)	P	Adjuvant TMZ monotherapy, was the only modifiable risk factor associated with MT of LGG, consistent with previous laboratory data of TMZ-induced hypermutation leading to MT. Despite, significant treatment bias, patients treated with adjuvant TMZ alone were more likely to be IDHmut1p/19qcodel (*P* < .001), which was also the subgroup at lowest risk of MT.

B, binary; C, categorical; D, delays MT; P, promotes MT.

### Tumour Volume and Expansion Velocity

Two parameters were identified recurrently in numerous studies, with varied numerical thresholds and levels of significance. The velocity of tumour growth or VDE appeared in four studies, with thresholds at ≥4 mm/y, ≥8 mm/y, and ≥12 cm/y. Incidentally discovered LGGs were found to grow 2.93 mm/y,^62^ while VDE ≥ 12 cm/y are infrequently encountered, setting two accepted thresholds at ≥4 mm and ≥8 mm/y, with the latter weighing worse prognostication. In addition, the presence of SVZ involvement appears to compound VDE and initial tumour volume appears to compound VDE.^62^

The significance of tumour volumetry appears in keeping with EOR studies examining PFS and OS in surgical series.^9,22,38,54^ For example, Smith et al reported that a minimum EOR of >90% of original volume is required to produce survival benefit and, more recently, absolute residual tumour volumes stratified between 0 cm^3^; 0.1–5.0 cm^3^ and >5.0 cm^3^ were associated with, consecutively decreasing survival benefit, for each group.^22^ Also, in a “near-randomised” trial, Roelz et al reported that survival benefit was recorded in patients with residual tumour volume of <15 cm^3^ and patients, in their series, where this threshold was not reached, fared similarly to the biopsy only group.^63^ Finally, accounting also for molecular subtyping, a recent large retrospective trial showed that any residual postoperative volume affected negatively the OS, even if the residual volume was only 0.1–5.0 cm^3^ regardless of the tumour's molecular profile.^64^ Nevertheless, these data refer to post-surgery volumes and not to the risk of MT in cohorts of patients that are followed up with serial imaging or post stereotactic biopsy.

However, multiple studies have shown that pre-operative volume, despite inconsistent definitions, in centimetres of maximum diameter or three-dimensional volume in millimetres, are distinct MT prognosticators. Numerical thresholds for the former definitions included ≥3 cm, and ≥5cm^9,35^ and for the latter >20 ml, >60 ml, and >100 ml.^20,33,36,53^ An older, 2002 EORTC study, based on 288 patients identified a threshold of ≥6c m as an unfavourable prognosticator.^65^ Not significantly deviating from this original observation, our analysis suggests a PTV of ≥5 cm was an independent MT prognosticator (*P* < .001)^37^; extrapolating the findings and converting radius to a volume with the formula V = (4/3)πr, indicates that a PTV ≥ 100 ml is a clear MT prognosticator, although the significance of lower thresholds cannot be disputed.

### Regional Blood Flow and Topography

The quantification and definition of increased rCBF compared to contralateral white matter varies across published studies but has been linked to MT in four studies. A threshold of rCBF = 1.742 appears to offer the best combination of sensitivity and specificity at 61.9% and 83.3%, respectively.^42^ The effect of rCBF on MT may be predictable, as for every SD of rCBF the risk of increased MT was estimated ×12.99.^40^ In addition, cortical and SVZ involvement were both independently associated with MT (*P* = .004 and *P* = .033, respectively^21,33^).

### Clinical Features

Epileptic seizures, a common presentation in most patients with LGGs,^66^ has been identified as a positive prognosticator in a number of studies, including a multi-institutional observational study confirming delay of MT (*P* < .001).^33^ The grade of tumours appears to be inversely related to incidence of seizures with grade I tumours such as dysembryoblastic neuroepithelial tumours (DNET) and gangliogliomas having an incidence of 80–100%, LGGs 60–85%, and HGGs a lower rate of 40–60%.^67^ It is likely that the destructive rather than irritative growth process in HGGs confers lower epilepsy incidence. To that end, oligodendroglial histology, conferring better prognosis compared to astrocytic one, also is associated with higher risk of epilepsy.^68^ Alternatively, seizures may trigger the earlier identification of the tumour resulting in a greater lead time to MT.

### Tissue-based, Post-surgery Prognosticators

Following molecular analysis of surgical specimens, our review identified a series of seven negative prognosticators: in addition to previously well-described IDHwt, Ki 67 labelling index over-expression, and positive VEGF, the cancer-protecting TP53 gene, either overexpressed or mutated, is also significant negative prognosticator in LGGs (*P* < .03).^50^ In addition, the triple combination of IDH mutation/MGMT methylation/TP53 confers a negative prognostic effect. Interestingly, although IDH mutation is associated with longer OS, the triple mutation, had significantly higher hazard to MT compared to IDHwt (*P* = .0452).^52^ In addition, positive and negative prognostication conferred by 1p/19q co-deletion, were independently confirmed with no reversed assumptions.^18,20,37,53,54^

Interestingly TZM monotherapy appears to be associated with MT. This may be a selection bias, as TMZ is likely to be offered in assumed higher-risk patients. A hypothesis suggesting the acquisition of TZM-associated mutations in key amino-acids of mismatch repair (MMR) genes, resulting in the accumulation of hypermutations, has been proposed.^69,70^ Further data are required to clarify if this is a relevant phenomenon that requires consideration when planning oncological care.

### LGGs Scoring Systems

As the growth rates, biological behaviour, and malignant transformation points of LGGs contain paucity of data, a number of scoring systems attempted to stratify hazards of poorer outcomes and early interventions were proposed. In 2002, Pignatti and colleagues used an EORTC database to score LGGs in five categories: age ≥ 40 years; astrocytoma cytology; tumour diameter ≥ 6 cm; tumour crossing the midline and presence of neurological deficit.^65^ Six years later the San Francisco group based on a series of 281 patients harbouring LGGs introduced a four-factor system scoring age >50 years; KPS < 80; presumed eloquent location; and maximum diameter > 4 cm.^68^

The evolution of molecular data and neuroimaging modalities as well as the accumulated experience of the last two decades resulted in accumulation of valuable data in the literature that the current study attempted to analyse and critically interpret. Although it is not our aim to propose a new scoring system, future efforts may include the 10-factor list, with weighing based on quantitative thresholds. For example, VDE > 4 mm/y and > 8 mm/y would add one and two points respectively, as this factor is categorical and not binary. Similarly, PTV ≥ 65 ml and PTV ≥ 100 ml would add one and two points respectively. Conversely, the presence of epileptic seizures would detract one point. However, this would remain a theoretical frame, and its validation is beyond the aim of this systematic review.

Consequently, an initial, proposed classification scheme for LGGs MT risk stratification is included (Table 2), divided to before intervention (i.e., radiological diagnosis only) and post-intervention (i.e., post diagnostic biopsy). For examples for patient cohorts followed up with serial imaging, before intervention risk scores (BI-RS) can be stratified as low (1–2 points), intermediate (3–4 points), or high (5–6 points). When additional molecular information is available following tissue diagnosis, the after-intervention risk score (AI-RS) is expanded to include low (1–3), intermediate (4–6), or high (7–9) risk scores. Following interinstitutional collaborations it is expected that the RS will be further defined.

**Table 2.** Proposed Initial Classification System for Risk Stratification to MT

	Domain	Factor	Threshold	Points
Before intervention (i.e., radiological diagnosis only)	Volume	PTV	>65 ml	1
			>100 ml	2
	Velocity	VDE	>4 mm/y	1
			>8 mm/y	2
	Location	Cortex involvement	Yes No	1 0
	Perfusion	rCBF	>1.75	1
After intervention (i.e., biopsy)	Genomics	TP53 mutation	Yes	1
		TP53 Overexpression	Yes	0
		IDH- wt	Yes	1
		IDH-mutant	Yes	0
	Treatment	TMZ monotherapy	Yes	1

Proposed initial classification system for risk stratification to MT, with three resulting groups: Before intervention risk scores: 1–2, low; 3–4, intermediate; 5–6, high. After intervention risk scores: 1–3, low; 4–6, intermediate; 7–9, risk.

### Clinical Practice Guidelines

Currently, no practice guidelines exist as to the ideal management of low-grade gliomas. Two Cochrane totalling 4139 and 2764 citations concluded that *“physicians must approach each case individually and weigh the risks and benefits of each intervention until further evidence is available”*,^71,72^ and that the timing of intervention could be weighed against the estimated risks of MT. The current systematic review offers a quantitative and measurable prognosticator system.

Limitations of our review included inconsistencies in definitions of MT across some studies, measurements of tumour volume, and quality of outcome measurements, although our critical analysis attempted to reconciliate such inconsistencies, where possible. Additionally, some studies reported data from the same patient datasets,^20,33^ as such the reports may contain duplicated patients. Finally, the majority of the studies included lacked information on the glioma molecular subtype, limiting the analysis as the prognostic factors could not be analysed with respect to molecular subgroup. To that end, the definition of what constitutes as a LGG has also been redefined in light of the molecular era, which some older studies in this review failed to appreciate by not differentiating between astrocytoma, oligodendroglioma, and oligoastrocytoma, the latter of which is considered to be entirely redundant. The prognostic factors should be addressed with respect to molecular status in future studies.

## Conclusion

LGGs do not follow a linear, predictable growth plot line, but display rather exponential intervals of accelerated, aggressive biological behaviour. Even when imaging appears to confer assurance to a static lesion, the background molecular changes cannot be displayed by current imaging modalities. The current systematic review has introduced 43 measurable, distinct, categorised prognosticators with numerical thresholds, and critically reviewed and weighted their importance. Although not the aim of this review, the analysed prognosticators can form the basis of a future, comprehensive scoring system.

## Supplementary Material

vdab101_suppl_Supplementary_Materials

## Notes

**Authorship Statement**. Data extraction: ZS, GS; Data analysis: ZS, GH, GS; Writing: ZS, GH, GS; Table and Figures: ZS, GS.
