# Supplementary material for: Systematic review—Time to malignant transformation in low-grade gliomas: Predicting a catastrophic event with clinical, neuroimaging, and molecular markers
Source: Neurooncol Adv. 2021 Jul 27;3(1):vdab101. doi: 10.1093/noajnl/vdab101 (PMC8403481; doi:10.1093/noajnl/vdab101)
Supplement: vdab101_suppl_Supplementary_Materials [file vdab101_suppl_supplementary_materials.docx]

**SUPPLEMENTARY ONLINE MATERIAL**

**eMethods**. Full Search Protocols for Ovid MEDLINE, Embase and Cochrane Databases

**eFigure 1.** Criteria List for Assessing Methodological Quality

**eFigure 2.** PRIMSA flow diagram

**eFigure 3.** Results of the Methodological Assessment for Included Studies

**eTable 1**. Summary of 43 prognosticators of MT identified

**eMethods:** Full search protocols for Ovid MEDLINE, Embase and Cochrane Databases

**Database: Ovid MEDLINE(R) and Epub Ahead of Print, In-Process & Other Non-Indexed Citations and Daily <1946 to April 28, 2021>**

1 glioma/ or astrocytoma/ or oligodendroglioma/

2 (glioma$ or astrocytoma$ or oligodendroglioma$).mp.

3 1 or 2

4 Cell Transformation, Neoplastic/

5 (malignant transformation or malignant degeneration or malignant progression).mp.

6 disease progression/

7 (disease adj3 progress$).mp.

8 neoplastic cell transformation.mp.

9 4 or 5 or 6 or 7 or 8

10 3 and 9

11 risk/ or risk factors/

12 biomarkers/ or biomarkers, tumor/ or genetic markers/

13 Prognosis/

14 risk$1.tw.

15 (marker$ or biomarker$ or factor$).mp.

16 prognosis.mp.

17 11 or 12 or 13 or 14 or 15 or 16

18 10 and 17

19 Comment/ or Letter/ or Editorial/

20 (comment or letter or editorial).pt.

21 or/19-20

22 18 not 21

**Database: Embase <1974 to 2021 April 28 >**

1 glioma/ or astrocytoma/ or oligodendroglioma/

2 (glioma$ or astrocytoma$ or oligodendroglioma$).mp.

3 1 or 2

4 malignant transformation/

5 (malignant transformation or malignant degeneration or malignant progression).mp.

6 disease progression/

7 (disease adj3 progress$).mp.

8 neoplastic cell transformation.mp.

9 4 or 5 or 6 or 7 or 8

10 3 and 9

11 risk/ or risk factor/

12 marker/

13 molecular marker/

14 genetic marker/

15 Prognosis/

16 risk$1.tw.

17 (marker$ or biomarker$ or factor$).mp.

18 prognosis.mp.

19 11 or 12 or 13 or 14 or 15 or 16 or 17 or 18

20 10 and 19

21 Comment/ or Letter/ or Editorial/

22 (comment or letter or editorial).pt.

23 21 or 22

24 20 not 23

**Cochrane Database of Systematic Reviews**

#1 MeSH descriptor: [Glioma] this term only

#2 MeSH descriptor: [Astrocytoma] this term only

#3 MeSH descriptor: [Oligodendroglioma] this term only

#4 glioma* or astrocytoma* or oligodendroglioma*

#5 #1 or #2 or #3 or #4

#6 MeSH descriptor: [Cell Transformation, Neoplastic] this term only

#7 “malignant transformation” or “malignant degeneration” or “malignant progression”

#8 MeSH descriptor: [Disease Progression] this term only

#9 disease near/3 progress*

#10 "neoplastic cell transformation"

#11 #6 or #7 or #8 or #9 or #10

#12 #5 and #11

#13 MeSH descriptor: [Risk] this term only

#14 MeSH descriptor: [Risk Factors] this term only

#15 MeSH descriptor: [Biomarkers] this term only

#16 MeSH descriptor: [Biomarkers, Tumor] this term only

#17 MeSH descriptor: [Genetic Markers] this term only

#18 MeSH descriptor: [Prognosis] this term only

#19 risk*

#20 prognosis

#21 #13 or #14 or #15 or #16 or #17 or #18 or #19 or #20

#22 #12 and #21

#23 #13 or #14 or #15 or #16 or #17 or #18 or #19

#24 #12 and #23

| 1.1 **Study participation** |
| --- |
| A. Description of study population |
| B. Description of inclusion and exclusion criteria |
| C. Description of baseline study population |
| 1.2 **Study attrition, follow-up (extent and length)** |
| D. Information about response rate of study participants |
| E. Dropouts/loss to follow-up ≤20% |
| F. Information about completers versus loss to follow-up/dropouts |
| 1.3 **Prognostic factors measurement** |
| G. Clearly defined description of the prognostic factor with valid method of measurement |
| H. The method and setting of measurement of prognostic factor is the same for all study participants |
| I. An adequate proportion of the study sample has complete data for the prognostic factor |
| J. Continuous variables are reported, or appropriate cut points are used |
| 1.4 **Outcome measurement** |
| K. Clearly defined outcome of MT with a valid and reliable method of outcome measurement |
| 1.5 **Confounding measurement and account** |
| L. Important confounders measured |
| M. Valid and reliable measurement of confounders |
| N. Appropriate accounting for confounding |
| 1.6 **Analysis** |
| O. Appropriate analysis techniques |
| P. Frequencies of most important prognostic factors |
| Q. Frequencies of most important outcome |

**eFigure 1:** Criteria List for Assessing Methodological Quality

**eFigure 2.** PRIMSA flow diagram

Records identified through database search
Embase: 2968

MEDLINE: 2954

Cochrane: 87

(n= 6009)

Additional records identified through other sources
(n = 1)

Records after duplicates removed
(n = 4314) + 1

Records screened by abstract and title for potential eligibility
(n = 4314) +1

Records excluded
(n = 4637)

Full-text articles assessed for eligibility
(n = 323) +1

Full-text articles excluded
(n = 290) + 1

Reasons for exclusion:

126 Definition of MT

unspecified/inconsistent

with inclusion criteria

46 Overall survival only

34 Not LGG

33 Not related to MT

13 Paediatric cohort

8 Pilocytic/ganglioglioma

8 No statistics

6 Non-English language

7 Full text not found

6 Review/ case report

4 Animal/cellular study

Studies included in qualitative synthesis
(n =33)

Studies included in quantitative synthesis
(n = 33)

**eFigure 3:** Results of the Methodological Assessment for Included Studies

| **Study** | **Study Participation** | **Study Attrition (Follow-up)** | **Prognostic Factor** | **Outcome** | **Confounding Factor** | **Analysis** | **Quality** |
| --- | --- | --- | --- | --- | --- | --- | --- |
| Zeng et al  (2021) | Low | Low | Low | Low | Low | Low | High |
| Rossi et al  (2021) | Low | Low | Low | Low | Low | Low | High |
| Tom et al (2019) | Low | Low | Low | Low | Low | Low | High |
| Morshed et al (2019) | Low | Low | Low | Low | Low | Low | High |
| Jansen et al (2019) | Low | Low | Low | Low | Moderate | Low | High |
| Jaber et al (2019) | Low | Low | Low | Low | Low | Low | High |
| Fukuya et al (2019) | Low | Low | Low | Low | High | Low | Low |
| Wen et al (2018) | Low | Low | Low | Low | Low | Low | High |
| Heo et al (2017) | Low | Low | Moderate | Low | Low | Low | High |
| Eseonu et al (2017) | Low | Low | Low | Low | Low | Low | High |
| Leu et al (2016) | Low | High | Moderate | Low | Low | High | Low |
| Pallud et al (2014) | Low | Low | Low | Low | Low | Low | High |
| Goze et al (2014) | Low | Low | Low | Low | Low | Low | High |
| Gousias et al (2014) | Low | Low | Low | Low | Low | Low | High |
| Pallud et al (2013) | Low | Low | Low | Low | Low | Low | High |
| Majchrzak et al (2012) | Low | Low | Low | Low | Low | Low | High |
| Juratli et al (2012) | Low | Low | Moderate | Low | Low | Low | High |
| Jakola et al (2012) | Low | Low | Low | Low | Low | Low | High |
| Ius et al (2012) | Low | Low | Low | Low | Low | Low | High |
| Hlaihel et al (2010) | Low | Low | Low | Low | Low | Moderate | High |
| Chaichana et al (2010) | Low | Low | Low | Low | Low | Low | High |
| Yue et al (2009) | Low | Low | Low | Low | High | Moderate | Low |
| Brasil Caseiras et al (2009) | Low | Low | Low | Low | Moderate | Low | High |
| Smith et al (2008) | Low | Low | Low | Low | Low | Low | High |
| Hattingen et al (2008) | Low | Low | Moderate | Low | High | Low | Low |
| Floeth et al (2007) | Low | Low | Low | Low | Moderate | Low | High |
| Mariani et al (2006) | Low | Low | Low | Low | Low | Low | High |
| Kreth et al (2006) | Low | Low | Low | Low | Low | Low | High |
| Stander et al (2004) | Low | Low | Low | Low | Low | Low | High |
| Ishii et al (1999) | Low | Low | Low | Low | Low | Low | High |
| Abdulrauf et al (1998) | Low | Low | Low | Low | Low | Low | High |
| Kreth et al (1997) | Low | Low | Low | Low | Low | Low | High |
| Chozick et al (1994) | Low | Low | Low | Low | Low | Low | High |

A study was rated for each of the 6 potential biases as having low (Y,YYY, YYYY, YYYU, NYYY, NYYU), moderate (U, YUU, NYUU, NYYU, NNYY, NNYU), or high (N, NNU, NNUU, NNNY, NNNU, NNNN) risk of bias per domain.

**eTable 1:** Summary of the 43 prognosticators for MT identified

| **DOMAIN** | **PROGNOSTICATOR** | **THRESHOLD(S) IN KEY STUDIES** | **EFFECT ON MT** | **STATISTICAL SIGNIFICANCE** | **COMMENTS** |
| --- | --- | --- | --- | --- | --- |
| Clinical | 1. Epilepsy | Presence/persistence ^1,2^ | D | P<0.001; P=0.011 | Large retrospective multi-institutional observational study with 1509 patients, history of epileptic seizures at diagnosis was a statistically significant (p <0.001), independent prognostic factor for delayed MT ^1^. |
|  | 1. KPS | ≥90 ^2^ | D | P = 0.005 |  |
|  |  | ≤70 ^1,3^ | P | P=0.032; P<0.05 |  |
|  | 1. Age | >45 years ^1,3^;  >35 years ^4^ | P | P=0.038;  P=0.05 |  |
|  | 1. Gender | Male ^1,5^ | P | P=0.001; P=0.009 |  |
|  | 1. ICP | Raised ^1,6^ | P | P=0.024; P=0.016 | Raised ICP was noted in 188/1509 patients in one study ^1^ and in 2/109 patients in another ^6^, both reported a negative prognostic effect. |
|  | 1. Neurological deficit | Presence ^1,2^ | P | P=0.01; P<0.001 |  |
|  | 1. Duration of symptoms | >2 years ^2^ | P | P=0.022 |  |
| Radiological/  imaging | 1. Circumscribed tumour | Presence ^7^ | D | P<0.0001 |  |
|  | 1. Local recurrence location | Presence ^8^ | D | P=0.0035 |  |
|  | 1. rCBV | >1.75 ^9^  High ^10-12^ | P | P=0.035  P=0.063; P=0.01; P=0.0347 | In one study (n=63), rCBV value of 1.742 was found to be of optimal sensitivity (61.9%) and specificity (83.3%) in differentiating the group with progression from the progression-free group ^9^.  A study of 34 patients found the risk of MT was 1.73x higher per SD of rCBV at study entry (P= 0.01; SD = 0.6) ^11^.  A study involving 21 patients showed that mean and maximum rCBV values were significantly higher in the transformers group compared with the non-transformers group, and rCBV >2 predicted MT had a sensitivity and specificity of 40% and 69%, respectively. The PPV and NPV were 29% and 79%, respectively ^12^. |
|  | 1. VDE | ≥8mm/year ^6,13,14^;  >3mm/year ^12^ | P | P<0.001; P<0.001; P=0.001;  P=0.0279 | A study involving 380 patients found that MPFS was significantly longer in the subgroup with VDE <8mm/year (median, 103 months; mean, 119.2 months; range, 1 – 253 months) than in the subgroup with VDE ≥8mm/year (median, 35 months; mean, 41.4 months; range, 2 – 206 months; P<0.001) ^13^.  In a study of 168 patients, VDE ≥ 4 mm/year, VDE ≥ 8 mm/year and VDE ≥ 12 mm/year were all independently associated with shorter MFS (P<0.001 for all) ^14^.  A study with 131 patients found that MFS was significantly longer in the VDE<8mm/year subgroup (median, 149 months; mean, 142) than in the VDE ≥8mm/year subgroup (median, 46 months; mean, 56.2; P<0.001) ^6^.  In 19 patients, VDE >3 mm/year was associated with MT with sensitivity and specificity of 80% and 86%, respectively, and PPV and NPV values of 67% and 92%, respectively ^12^. |
|  | 1. △VT2T1 | >30cm^3^ ^15^ | P | P<0.0001 | In a study involving 190 patients both △VT2T1 ≥ 30cm^3^ and an increased △VT2T1 value, modelled as a continuous variable, was associated with shorter MPFS (P<0.0001) ^15^. |
|  | 1. Contrast on MRI/CT | Presence ^1,2,13,16,17^ | P | P<0.05; P=0.011; P<0.001; P=0.013; P=0.014 |  |
|  | 1. ADC (apparent diffusion coefficient) | Low ^10^ | P | P=0.005 | Low ADC indicates hypo intensity on ADC maps compared to the adjacent brain parenchyma.  Quantitative analysis on ADC was not feasible since multi-parametric MR protocols have been changed during the last 10 years ^10^. |
|  | 1. Normalised tCr | High ^16^ | P | P=0.016 | Study of 45 patients suggests that LGGs with tCr values <1.0 may show later MT than gliomas with normal or increased tCr values ^16^. |
|  | 1. Mean and maximal choline/creatine ratio | High ^12^ | P | P=0.0003; P<0.0001 | In 21 patients a choline/creatine ratio >2.4, was associated with a MT risk of 83% and a mean delay of 15.4 months. Conversely, patients with a choline/creatine ratio <2.4 had a 100% chance of not transforming during the same period ^12^. |
|  | 1. fluorescence with 5-ALA | Presence ^18^ | P | P=0.01 | MTFS was shorter in fluorescing vs. non-fluorescing tumors (43.0, 27.5-58.5 vs 64.6, 57.7-71.5 months) (P=0.015) ^18^. |
|  | 1. Tumour growth in 6 months | Greater ^11^ | P | P = 0.001 | The risk of MT was 3.63x higher per each additional SD of tumour growth within 6 months (95% CI: 1.75, 7.49; P = 0.001; SD = 7.1 mL) ^11^. |
|  | 1. Mean ^18^F-FET uptake | >1.1 ^7^ | P | P=0.006 |  |
|  | 1. Early tumour recurrence | Presence ^8^ | P | P = 0.0022 | In this study early recurrences were defined as within 2 years of primary surgery ^8^. |
|  | 1. Speed of radiological progression | Fast ^8^ | P | P = 0.0011 | In the study, fast was defined as the interval from initial imaging changes to definitive radiological diagnosis being ≤6 months) ^8^. |
| Molecular/  biological | 1. 1p/19q status | Codeletion ^13,19,20^ | D | P=0.048;  P=0.021;  P=0.001 | In a study of 51 patients, 1p19q codeletion independently predicted longer MFS (P=0.048), whilst its absence, with or without the IDH1 mutation, was significantly associated with worse MFS (P=0.047 and P=0.013) ^20^.  A retrospective study with 197 patients, showed 1p19q codeletion to be a significant and independent prognostic factor for delayed MT (P=0.021) ^13^.  In a bi-centric retrospective study involving 110 patients, the strongest prognostic factor for MT was an oligodengoglial phenotype (IDHmut/codel) with significantly later MT than astrocytomas (IDHmut and IDHwt; P=0.013)^19^. |
|  |  | Intact ^5,20,21^ | P | P=0.013;  P=0.009;  P<0.05 | In a retrospective cohort study of 486 patients IDHmut1p/19qintact tumours were significantly more likely to undergo MT than IDHmut1p/19qcodel tumours (P=0.009), with the 5-year estimates of freedom from MT being 92% for IDHmut1p/19qcodel and 86% IDHmut1p/19qintact tumours ^5^. |
|  | 1. IDH1 wt | Presence ^5,6,18^ | P | P=0.019; P<0.001; P=0.043 | In a retrospective cohort study of 486 patients IDHwt tumours were significantly more likely to undergo MT than IDHmut1p/19qcodel tumours (P<0.001), with the 5-year estimates of freedom from MT being 82% for IDHwt and 86% IDHmut1p/19qintact tumours ^5^.  In a study of 131 patients, Lack of IDH1 mutation was independently and significantly associated with shortened MFS (P=0.019) ^6^. |
|  | 1. IDH-mutated/*MGMT*-methylated/TP53- positive | Presence ^22^ | P | P = 0.0452 | In 105 patients, tumours with the triple combination of IDH-mutated/MGMT-methylated/TP53-positive, had a significantly higher hazard for MT than IDHwt tumours (P = 0.0452) ^22^. |
|  | 1. P53/TP53 overexpression | Presence ^23,24^ | P | P<0.05; P<0.0001 | Positive TP53 mutation status (but not P53 overexpression) was the lone risk factor with respect to MT in a series with 159 patients ^25^.  In 36 patients, TP53 was strongly associated with MT. Nine of 14 tumors (75%) harboring TP53 mutations showed MT within 12±75 months (median 37 months), compared to 9/22 tumors (41%) wild type TP53 tumours ^26^. |
|  | 1. TP53 mutation | Presence ^25,26^ | P | P<0.03; P=0.0344 |  |
|  | 1. VEGF staining | Positive ^3^ | P | P=0.002 |  |
|  | 1. Microvessel density | >7 ^3^ | P | P=0.001 | The 5-year probability of MT was 72% (95% CI: 54–90%) for the 29 patients with more than seven vessels compared with 28% (95% CI: 13–43%) for the 45 with seven or fewer microvessels ^3^. |
|  | 1. Ki 67 labelling index overexpression | Presence ^23^ | P | P<0.05 |  |
| Tumour volumetry | 1. Initial TV/size | >3cm ^3,27^;  >5cm ^2,5^;  >20ml ^4^;  >60mL ^21^;  ≥100cm^3 1,13^;  Larger (without specific threshold) ^9,15,28-30^ | P | P=0.03; P<0.05  P=0.047; P<0.001;  P=0.01;  P<0.001;  P=0.008; P=0.007;  P=0.002; P=0.004; P=0.023; P=0.001; P=0.022 | In a retrospective study of 353 patients, tumour size ≥5cm was a statistically significant, independent prognosticator for MT (P<0.001), with the risk being 3.5-fold higher for tumours of this size ^5^.  In 148 patients, tumours >5cm (preoperative maximal diameter) was significantly associated with worsened MFS (P=0.047) ^2^.  Volume of ≥100 ml was independently and statistically associated with shortened MFS in 380 patients (P=0.008)^13^.  In 1509 patients, tumour volume of ≥100 ml was an independent factor for worsened MFS (P=0.007) ^1^. |
|  | 1. Post-operative TV | >30ml ^21^;  Larger (without specific threshold) ^15,28^ | P | P<0.01;  P=0.03; P<0.0001 |  |
|  | 1. Surgery | Larger %EOR ^15,29^;  EOR>90% ^8^;  GTR ^1,2,6,19,27,31^;  1st line surgical resection (vs. surveillance) ^13^;  Partial resection (vs. biopsy) ^1,2^;  Subtotal resection (vs. biopsy) ^1^ | D | P=0.005; P<0.0001;  P=0.0395;  P=0.05; P=0.025; P<0.001; P<0.001;  P=0.031;  P<0.001  P<0.001  P<0.001; P=0.015  P<0.001 |  |
|  |  | Postoperative tumour volume ≤ 5 mL (vs. >5 mL) ^32^ | D | P<0.01 |  |
|  |  | Watchful waiting (vs. resection) ^33^;  Smaller EOR ^9^ | P | P=0.02  P=0.02 |  |
| Topological/  anatomical | 1. SVZ involvement | Presence ^(21)^ | P | P=0.033 |  |
|  | 1. Parietal tumour | Presence ^6^ | P | P=0.019 |  |
|  | 1. Cortical involvement | Presence ^1^ | P | P=0.004 | The presence of cortical involvement was an independent negative prognosticator for MT in a retrospective study of 1509 patients (P=0.004) ^1^. |
|  | 1. Eloquent tumour | Presence ^2,29^ | P | P=0.006; P<0.001 |  |
|  | 1. Multilobar involvement | Presence ^21,30^ | P | P<0.01; P=0.048 |  |
| Histological | 1. Fibrillary astrocytoma | Presence ^15,27^ | P | P=0.04; P=0.003 |  |
|  | 1. Pure astrocytoma | Presence ^9,21^ | P | P=0.03; P<0.05 |  |
|  | 1. Gemistocytic astrocytoma | Presence ^10,21^ | P | P=0.015; P<0.01 | The gemistocytic astrocytoma group showed a significantly shorter median time to MT than the non- gemistocytic group (54 months vs 65.1 months; P = 0.035) ^10^. |
| Treatment | 1. CT | Presence ^1^ | D | P<0.001 |  |
|  |  | Monotherapy (vs. CT+ RT) ^5^ | P | P=0.008 |  |
|  | 1. RT | Presence ^1,15^ | D | P<0.001; P=0.032 |  |
|  | 1. Adjuvant CT-RT | Presence ^19^ | D | P=0.0001 | Adjuvant TMZ monotherapy, was the only modifiable risk factor associated with MT of LGG, consistent with previous laboratory data of TMZ-induced hypermutation leading to MT.  Despite, significant treatment bias, patients treated with adjuvant TMZ alone were more likely to be IDHmut1p/19qcodel (P<0.001), which was also the subgroup at lowest risk of MT ^19^. |

Abbreviations: D, delays; P, promotes; MPFS, malignant progression free survival; PPV, positive predictive value; NPV, negative predictive value

**REFERENCES: ONLINE SUPPLEMENT**

1. Pallud J, Audureau E, Blonski M, et al. Epileptic seizures in diffuse low-grade gliomas in adults. *Brain : a journal of neurology.* 2014;137(Pt 2):449-462.

2. Gousias K, Schramm J, Simon M. Extent of resection and survival in supratentorial infiltrative low-grade gliomas: analysis of and adjustment for treatment bias. *Acta neurochirurgica.* 2014;156(2):327-337.

3. Abdulrauf SI, Edvardsen K, Ho KL, Yang XY, Rock JP, Rosenblum ML. Vascular endothelial growth factor expression and vascular density as prognostic markers of survival in patients with low-grade astrocytoma. *Journal of neurosurgery.* 1998;88(3):513-520.

4. Kreth FW, Faist M, Grau S, Ostertag CB. Interstitial 125I radiosurgery of supratentorial de novo WHO Grade 2 astrocytoma and oligoastrocytoma in adults: long-term results and prognostic factors. *Cancer.* 2006;106(6):1372-1381.

5. Tom MC, Park DYJ, Yang K, et al. Malignant Transformation of Molecularly Classified Adult Low-Grade Glioma. *International journal of radiation oncology, biology, physics.* 2019.

6. Goze C, Blonski M, Le Maistre G, et al. Imaging growth and isocitrate dehydrogenase 1 mutation are independent predictors for diffuse low-grade gliomas. *Neuro-oncology.* 2014;16(8):1100-1109.

7. Floeth FW, Pauleit D, Sabel M, et al. Prognostic value of O-(2-18F-fluoroethyl)-L-tyrosine PET and MRI in low-grade glioma. *Journal of nuclear medicine : official publication, Society of Nuclear Medicine.* 2007;48(4):519-527.

8. Fukuya Y, Ikuta S, Maruyama T, et al. Tumor recurrence patterns after surgical resection of intracranial low-grade gliomas. *Journal of neuro-oncology.* 2019;144(3):519-528.

9. Majchrzak K, Kaspera W, Bobek-Billewicz B, et al. The assessment of prognostic factors in surgical treatment of low-grade gliomas: a prospective study. *Clinical neurology and neurosurgery.* 2012;114(8):1135-1144.

10. Heo YJ, Park JE, Kim HS, et al. Prognostic relevance of gemistocytic grade II astrocytoma: gemistocytic component and MR imaging features compared to non-gemistocytic grade II astrocytoma. *European radiology.* 2017;27(7):3022-3032.

11. Brasil Caseiras G, Ciccarelli O, Altmann DR, et al. Low-grade gliomas: six-month tumor growth predicts patient outcome better than admission tumor volume, relative cerebral blood volume, and apparent diffusion coefficient. *Radiology.* 2009;253(2):505-512.

12. Hlaihel C, Guilloton L, Guyotat J, Streichenberger N, Honnorat J, Cotton F. Predictive value of multimodality MRI using conventional, perfusion, and spectroscopy MR in anaplastic transformation of low-grade oligodendrogliomas. *Journal of neuro-oncology.* 2010;97(1):73-80.

13. Pallud J, Blonski M, Mandonnet E, et al. Velocity of tumor spontaneous expansion predicts long-term outcomes for diffuse low-grade gliomas. *Neuro-oncology.* 2013;15(5):595-606.

14. Wen B, Fu F, Hu L, Cai Q, Xie J. Subventricular zone predicts high velocity of tumor expansion and poor clinical outcome in patients with low grade astrocytoma. *Clinical neurology and neurosurgery.* 2018;168:12-17.

15. Ius T, Isola M, Budai R, et al. Low-grade glioma surgery in eloquent areas: Volumetric analysis of extent of resection and its impact on overall survival. A single-institution experience in 190 patients - Clinical article. *Journal of Neurosurgery.* 2012;117(6):1039-1052.

16. Hattingen E, Raab P, Franz K, et al. Prognostic value of choline and creatine in WHO grade II gliomas. *Neuroradiology.* 2008;50(9):759-767.

17. Kreth FW, Faist M, Rossner R, Volk B, Ostertag CB. Supratentorial World Health Organization Grade 2 astrocytomas and oligoastrocytomas. A new pattern of prognostic factors. *Cancer.* 1997;79(2):370-379.

18. Jaber M, Ewelt C, Wolfer J, et al. Is Visible Aminolevulinic Acid-Induced Fluorescence an Independent Biomarker for Prognosis in Histologically Confirmed (World Health Organization 2016) Low-Grade Gliomas? *Neurosurgery.* 2019;84(6):1214-1224.

19. Jansen E, Hamisch C, Ruess D, et al. Observation after surgery for low grade glioma: long-term outcome in the light of the 2016 WHO classification. *Journal of neuro-oncology.* 2019.

20. Eseonu CI, Eguia F, ReFaey K, et al. Comparative volumetric analysis of the extent of resection of molecularly and histologically distinct low grade gliomas and its role on survival. *Journal of neuro-oncology.* 2017;134(1):65-74.

21. Mariani L, Deiana G, Vassella E, et al. Loss of heterozygosity 1p36 and 19q13 is a prognostic factor for overall survival in patients with diffuse WHO grade 2 gliomas treated without chemotherapy. *Journal of clinical oncology : official journal of the American Society of Clinical Oncology.* 2006;24(29):4758-4763.

22. Leu S, von Felten S, Frank S, Boulay J-L, Mariani L. IDH mutation is associated with higher risk of malignant transformation in low-grade glioma. *Journal of neuro-oncology.* 2016;127(2):363-372.

23. Yue WY, Yu SH, Zhao SG, Chen ZP. Molecular markers relating to malignant progression in Grade II astrocytoma: Laboratory investigation. *Journal of Neurosurgery.* 2009;110(4):709-714.

24. Chozick BS, Pezzullo JC, Epstein MH, Finch PW, Raffel C, Rutka JT. Prognostic implications of p53 overexpression in supratentorial astrocytic tumors. *Neurosurgery.* 1994;35(5):831-838.

25. Stander M, Peraud A, Leroch B, Kreth FW. Prognostic impact of TP53 mutation status for adult patients with supratentorial World Health Organization Grade II astrocytoma or oligoastrocytoma: a long-term analysis. *Cancer.* 2004;101(5):1028-1035.

26. Ishii N, Tada M, Hamou MF, et al. Cells with TP53 mutations in low grade astrocytic tumors evolve clonally to malignancy and are an unfavorable prognostic factor. *Oncogene.* 1999;18(43):5870-5878.

27. Chaichana KL, McGirt MJ, Laterra J, Olivi A, Quinones-Hinojosa A. Recurrence and malignant degeneration after resection of adult hemispheric low-grade gliomas: Clinical article. *Journal of Neurosurgery.* 2010;112(1):10-17.

28. Morshed RA, Han SJ, Hervey-Jumper SL, et al. Molecular features and clinical outcomes in surgically treated low-grade diffuse gliomas in patients over the age of 60. *Journal of neuro-oncology.* 2019;141(2):383-391.

29. Smith JS, Chang EF, Lamborn KR, et al. Role of extent of resection in the long-term outcome of low-grade hemispheric gliomas. *Journal of clinical oncology : official journal of the American Society of Clinical Oncology.* 2008;26(8):1338-1345.

30. Juratli TA, Kirsch M, Robel K, et al. IDH mutations as an early and consistent marker in low-grade astrocytomas WHO grade II and their consecutive secondary high-grade gliomas. *Journal of neuro-oncology.* 2012;108(3):403-410.

31. Zeng L, Mei Q, Li H, Ke C, Yu J, Chen J. A survival analysis of surgically treated incidental low-grade glioma patients. *Scientific Reports.* 2021;11(1):8522.

32. Rossi M, Gay L, Ambrogi F, et al. Association of supratotal resection with progression-free survival, malignant transformation, and overall survival in lower-grade gliomas. *Neuro-Oncology.* 2020;23(5):812-826.

33. Jakola AS, Myrmel KS, Kloster R, et al. Comparison of a strategy favoring early surgical resection vs a strategy favoring watchful waiting in low-grade gliomas. *JAMA - Journal of the American Medical Association.* 2012;308(18):1881-1888.
